# Collective decision making and social interaction rules in mixed-species flocks of songbirds

**DOI:** 10.1016/j.anbehav.2014.07.008

**Published:** 2014-09

**Authors:** Damien R. Farine, Lucy M. Aplin, Colin J. Garroway, Richard P. Mann, Ben C. Sheldon

**Affiliations:** a Edward Grey Institute of Field Ornithology, Department of Zoology, Oxford, U.K.; b Department of Anthropology, University of California Davis, Davis, CA, U.S.A.; c Smithsonian Tropical Research Institute, Ancon, Panama; d Research School of Biology (Division of Ecology, Evolution and Genetics), Australian National University, Acton, ACT, Australia; e Department of Mathematics, University of Uppsala, Uppsala, Sweden

**Keywords:** collective behaviour, *Cyanistes caeruleus*, decision making, interspecific interaction, mixed-species flocking, Paridae, *Parus major*, social information use

## Abstract

Associations in mixed-species foraging groups are common in animals, yet have rarely been explored in the context of collective behaviour. Despite many investigations into the social and ecological conditions under which individuals should form groups, we still know little about the specific behavioural rules that individuals adopt in these contexts, or whether these can be generalized to heterospecifics. Here, we studied collective behaviour in flocks in a community of five species of woodland passerine birds. We adopted an automated data collection protocol, involving visits by RFID-tagged birds to feeding stations equipped with antennae, over two winters, recording 91 576 feeding events by 1904 individuals. We demonstrated highly synchronized feeding behaviour within patches, with birds moving towards areas of the patch with the largest proportion of the flock. Using a model of collective decision making, we then explored the underlying decision rule birds may be using when foraging in mixed-species flocks. The model tested whether birds used a different decision rule for conspecifics and heterospecifics, and whether the rules used by individuals of different species varied. We found that species differed in their response to the distribution of conspecifics and heterospecifics across foraging patches. However, simulating decisions using the different rules, which reproduced our data well, suggested that the outcome of using different decision rules by each species resulted in qualitatively similar overall patterns of movement. It is possible that the decision rules each species uses may be adjusted to variation in mean species abundance in order for individuals to maintain the same overall flock-level response. This is likely to be important for maintaining coordinated behaviour across species, and to result in quick and adaptive flock responses to food resources that are patchily distributed in space and time.

## Highlights

• We investigate the movement decisions by PIT-tagged birds in mixed-species flocks.
• Individuals had a strong tendency to move towards others when foraging in patches.
• We fit a model to infer decision-making rules used by birds in foraging flocks.
• Birds used rules adapted to the abundance of conspecifics and heterospecifics.
• A simple rule of attraction may be a flexible response to ecological conditions.

Group living is an integral part of the life history of many animals, providing benefits to individual participants by reducing predation risk (Cresswell and Quinn, 2004, Hamilton, 1971, Ioannou et al., 2012, Krause and Ruxton, 2002), facilitating information transfer (Couzin, 2009) and improving decision making (Sumpter et al., 2008, Ward et al., 2011, Ward et al., 2012, Ward et al., 2008). However, social living may also be costly, as it can increase resource competition (Dhondt, 2012, Krause and Ruxton, 2002), and exposure to parasites and disease (Krause & Ruxton, 2002). One common strategy to reduce competition while maintaining antipredation benefits is to join mixed-species groups (Greenberg, 2000, Harrison and Whitehouse, 2011, Krause and Ruxton, 2002). By associating with ecologically similar, but not identical, species, individuals may potentially be able to continue acquiring relevant benefits such as safety from shared predators (Sridhar, Beauchamp, & Shanker, 2009) and information about the environment (Seppanen, Forsman, Monkkonen, & Thomson, 2007), while reducing niche overlap (Greenberg, 2000, Harrison and Whitehouse, 2011, Krause and Ruxton, 2002). If this hypothesis is true, we predict that, given a choice of where to forage within a patch, moving individuals should choose areas of high density, regardless of species. However, the strength of social attraction may vary, reflecting individual and species differences in the balance of costs and benefits, or the need to maintain flock-level cohesion (Aplin, Farine, Mann, & Sheldon, 2014). To test these predictions, we investigated the flocking dynamics in a wild population of songbirds.

Studies of the social behaviour of monospecific groups have shown that strikingly complex patterns of movement and group behaviour can emerge from relatively simple social interactions between individuals (often referred to as collective animal behaviour; Ballerini et al., 2008, Buhl et al., 2006, Guttal and Couzin, 2010, Ioannou et al., 2012, Sumpter, 2006, Sumpter, 2010). These patterns can often be reproduced using simple algorithmic rules (Couzin and Krause, 2003, Herbert-Read et al., 2011, Katz et al., 2011, Sumpter, 2010). The emergence of complex grouping behaviour from simple social rules based upon attraction to, and repulsion from, nearby conspecifics (Arganda et al., 2012, Couzin and Krause, 2003, Herbert-Read et al., 2011, Katz et al., 2011, Pérez-Escudero and Polavieja, 2011, Pérez-Escudero et al., 2013, Sumpter, 2010) could apply equally to mixed-species groups (Farine et al., 2014, Jolles et al., 2013), such as in mixed schools of fishes (Hoare, Ruxton, Godin, & Krause, 2000), herds of ungulates (Fitzgibbon, 1990) or flocks of birds (Farine, 2013a, Farine et al., 2012, Farine and Milburn, 2013). As Morse (1970, p. 120) stated, ‘[group] formation depends upon positive responses by individuals to members of their own or other species’, where the positive response separates mixed-species groups from aggregations at a locally abundant resource (such as food or water).

One approach that has successfully linked individual decision rules to the biology of social groups is a combination of empirical data with mathematical models of decision making derived from theory (Sumpter, Mann, & Perna, 2012). Fitting models to empirical data has been used in order to determine the rules that maintain synchrony in birds (Ballerini et al., 2008), fish (Herbert-Read et al., 2011, Katz et al., 2011) and invertebrates (Ame, Halloy, Rivault, Detrain, & Deneubourg, 2006). Once a predictive model is generated, simulations can be used to make predictions about the adaptive function of these rules. For example, the aggregation rule used by cockroaches (Ame et al., 2006) was found to maximize individual fitness when simulated in agent-based models. As a result, this study suggested that temporary safe patches can emerge as a by-product of the dynamic self-organization by individuals responding to the distribution of others, even in a uniform landscape (Ame et al., 2006).

We recorded the movement decisions of individually marked birds participating in mixed-species flocks to investigate the social rules that drive the formation and maintenance of animal groups. (1) We investigated within-flock dynamics in order to determine whether birds moved towards others or away from them when foraging in food patches. (2) We then compared these patterns to a null model in order to determine how the observed pattern of movement differs from random. (3) We then fitted a Bayesian decision-making model (Arganda et al., 2012) that enabled us to determine (a) whether birds had different rules for conspecifics and heterospecifics, and (b) whether species varied in their use of conspecific and heterospecific interaction rules. (4) Finally, we used an agent-based model to determine whether inferred interaction rules could quantitatively reproduce the patterns we observed and to explore the properties of the decision-making rules that we inferred. In doing so, this study provides a benchmark for understanding the nature of mixed-species flocks using some recently developed approaches from computational biology.

## Methods

### Study Site and General Protocol

The study took place at Wytham Woods (51° 46′N, 1° 20′W), Oxfordshire, U.K. Great tits, *Parus major*, blue tits, *Cyanistes caeruleus*, marsh tits, *Peocile palustris*, coal tits, *Periparus ater*, and Eurasian nuthatches, *Sitta europaea*, were caught in mist nets using multi-access feeders regularly during the two winters in which the study took place. In addition, locally breeding birds and their offspring were caught in their nestboxes during the spring as part of long-term field studies in this population (Aplin et al., 2012, Farine and Lang, 2013). All individuals were fitted with a British Trust for Ornithology (BTO) metal leg ring and a plastic leg ring containing a uniquely coded PIT tag (IB Technology, Aylesbury, U.K.). We estimate that the proportion of the population fitted with PIT tags exceeded 90% at the time of the study (Aplin, Farine, et al., 2013), and we do not expect that untagged birds had much impact on our results. We conducted five replicates of the study in February 2011 and 15 replicates between December 2011 and February 2012. Replicates were placed throughout the woods, capturing the variation in population sizes driven by different understory habitat densities, and other habitat features. On some occasions, up to three replicates were running simultaneously; however, these were spaced at least 1 km apart and no individuals were detected at more than one replicate when replicates were operating simultaneously.

### Field Observations

**Figure 1:**
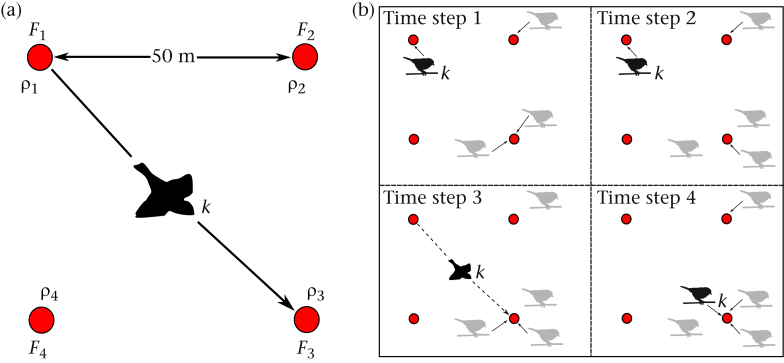
Overview of the experimental design and data collection. (a) Schematic of an experimental habitat patch. Individual *k* leaves feeder *F*_*i*_ with relative density ρ_*i*_ and arrives at feeder *F*_*j*_ with relative density ρ_*j*_. Here, *i* = 1 and *j* = 3. Birds are free to arrive and leave the patch at all times. (b) A toy example of the focal individual *k* (in black) and its flock in a patch. *k* is detected feeding on feeder *F*_1_ at *t* = 1–2, during which time the distribution of individuals across feeders *F*_1_–*F*_4_ is 0, 0.25, 0.75, 0, respectively (note that the focal individual is removed from influencing its own decision), hence ρ_*i*_ = 0. Individual *k* is then detected at *F*_3_ at *t* = 4, where ρ_*j*_ = 0.75.

At each replicate, we deployed a square of four identical feeders filled with unhusked sunflower seeds (henceforth a ‘patch’; Fig. 1a). Each feeder contained two access holes, both fitted with an antenna capable of reading the PIT tag fitted to birds as they land on the surface of the antenna (Francis Instruments, Cambridge, U.K.). We filled feeders with sunflower seed, which birds typically pick up by landing on the feeder and then fly to a nearby tree to process (see Supplementary movie), thereby minimizing interference competition (Aplin, Sheldon, & Morand-Ferron, 2013). Further, these feeders provide food at a constant rate thereby removing any effects of perceived resource depletion on foraging decisions (Stephens, Brown, & Ydenberg, 2007). These feeders also represented by far the most abundant food source available in the local patch, and the availability of nonfeeder options nearby were unlikely to have much impact on the behaviour of visiting birds. Eating seed in this fashion, birds did not form independent groups on each feeder, but maintained more natural flock formation in the nearby trees.

Feeders were placed 50 m apart, which is within visual and auditory range of other birds, but avoids the potential for individuals to feed on different feeders from the same perching location. To minimize differences in microhabitat features (presence of nearby habitat refuges) that are known to alter feeding behaviour (Dolby & Grubb, 2000), we moved some feeders inwards up to 5 m when setting out each patch. Patches were always contained within areas with uniform habitat density (but these could vary between patches). Each patch was set out and marked in the days preceding deployment. Feeders were then installed after dark the night before we started data logging to enable natural discovery of the patch. Patches were checked from day 2 onwards and removed once the food in any one feeder was fully depleted; if this did not happen the deployment was ended on the fourth day and data from that day were discarded. The antennae recorded the identity of all birds visiting the feeder, scanning for the presence of a tag every 1/16th of a second and logging one record per bird in each 15 s interval.

### Data Analysis

#### Overview

To infer the interaction rules that are used in mixed-species flocking, our analysis followed five steps.

(1) Individuals were detected moving between feeders within the patch. Each of these detections represented one within-flock movement decision (one data point in our analyses).

(2) We used Bayes' rule to calculate the probability of moving from one feeder to another conditioned on the distribution of individuals across the foraging patch. This provided us with an overview of the general movement patterns.

(3) We compared these movements to a null model in which birds moved simply as a function of feeder density, similar to the ideal free distribution. This enabled us to determine whether birds were moving towards or away from flockmates.

(4) We inferred a common decision-making rule by fitting the parameters of a Bayesian decision-making model using maximum-likelihood estimation. A strong relationship, given by a high value of the parameter *s* (see below), suggests individuals rely strongly on social information or are strongly attracted to others. The model was fitted separately to decisions made by blue tits and great tits, and we fitted separate *s* parameters for conspecifics and heterospecifics in order to determine whether birds used different rules for different components of their flocks.

(5) Finally, we used flock-level parameters from the observed data and the best-fitting model to simulate decisions. This allowed us to test whether the best-fitting decision rule we inferred can successfully replicate our data, and how the properties of decision making varied between species.

#### Details of analysis

**Figure A1:**
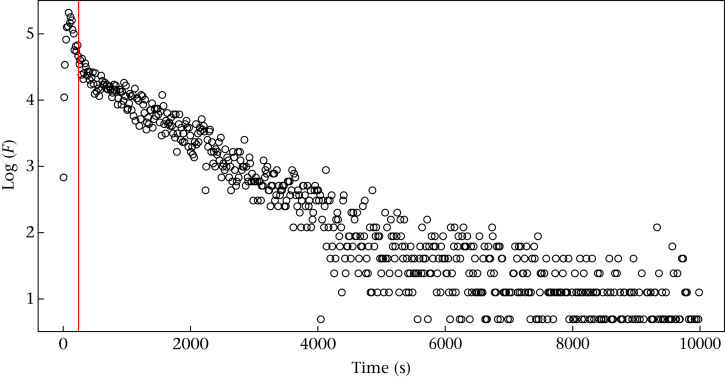
Logged frequency of delay times between observations of individuals made at different feeders. The vertical line (time = 240 s) represents the value used in the analyses.

(1) To identify movement decisions we combined the records from each of the four feeders into one data stream and extracted every occurrence of an individual moving between feeders within a patch (Fig. 1b). Individuals were defined as having remained within the patch if the gap between successive logged visits was no greater than 240 s. This value was based on the estimated inflection of the Poisson-distributed movement times (Appendix Fig. A1), representing the point where the distribution changes from the peak to the tail. Biologically, this point represents where repeated samples taken from the right-hand side of the distribution are more different (intervisit intervals are more likely to be different) than from the left-hand side (intravisit intervals are less likely to be different). For each movement event, we recorded which feeders the individual moved from and which it moved to, as well as the distribution of all other individuals in the patch at the time of each event.

(2) To determine the attraction or repulsion to others in their flock, we used Bayes' rule to calculate the probability of a movement between feeders (leaving one and arriving at another) conditioned on the relative proportion of individuals present on each feeder. The distribution of individuals across feeders was taken from detections on each feeder in the 30 s prior to departure or arrival (two 15 s time steps from our logging hardware, see Fig. 1b). The probability of an arrival *P*(*A*) at a feeder given a density *ρ* was then calculated using an equation from Mann (2011) and Pérez-Escudero and De Polavieja (2011):

(1) $$P\left(A|\text{ρ}\right)=\frac{P\left(\text{ρ}|A\right)P\left(A\right)}{P\left(\text{ρ}\right)}$$

where *P*(ρ) is the frequency (i.e. probability distribution) of densities ρ that were observed on all feeders (taken from all visits in the data), and *P*(*A*) is the prior probability of an arrival at a given feeder independent of proportion (which we fixed at *P*(*A*) = 0.25 since all feeders were of equal quality). *P*(ρ|*A*) is the observed frequency of a density ρ at the arrival feeder when an individual was detected moving (see Fig. 1). We also calculated the probability of leaving (*L*) conditioned on the density of individuals at the leaving feeder *P*(*L*|ρ) using the same equation.

(3) Because the probability of moving between sites is not independent of density, we generated a null probability of leaving and arriving conditioned on the density at the feeder, against which we could compare our results. We define this as the theoretical asocial prediction (TASP). In the TASP, individuals moved to (arrived at) feeders with a probability inverse to the density (*P*(ρ) ∝ 0.33ρ, given that by definition the choice is limited to three feeders), and moved from (left) feeders in proportion to the number of individuals at that feeder (*P*(ρ) ∝ ρ). Our TASP is important as it distinguishes randomly selecting individuals to make a move from a null model that randomly selects two feeders to move between. For example, if all individuals are at a feeder with ρ = 1, then any bird that moves must leave that feeder with a probability of 1. In contrast, a null model that randomly selects feeders with a fixed probability of leaving of 0.25 and arriving of 0.33 incorrectly models this relationship.

(4) To fit the Bayesian decision-making model we used a recently published model derived by Arganda et al. (2012):

(2) $$P\left(X_{i}|B\right)=\frac{1}{1+as^{-\left(n_{x}-k\sum_{i\neq x}n_{i}\right)}}$$

**Figure 2:**
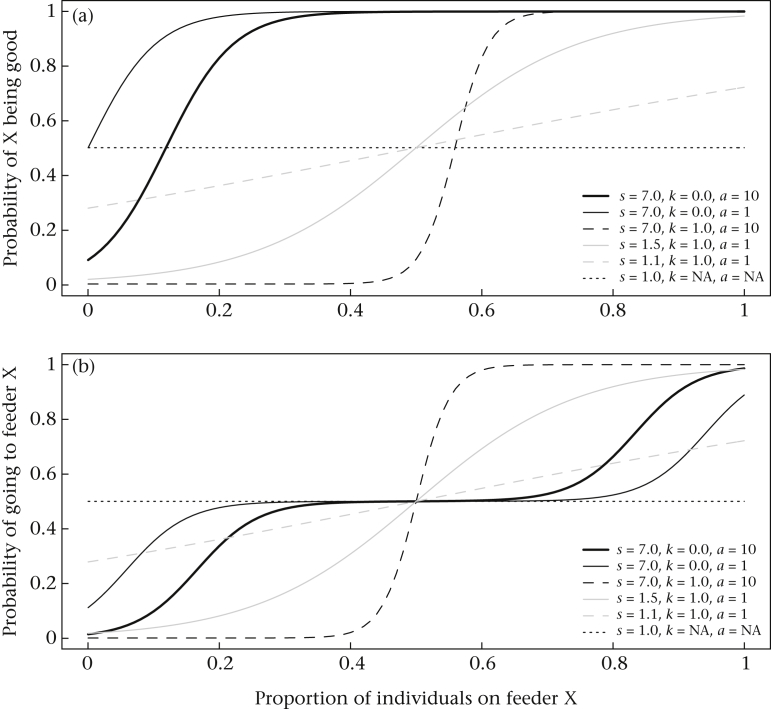
Overview of the relationship between the proportion of individuals on a site and (a) the probability of that site being identified as good and (b) the probability of choosing that site in a two-site decision, under the decision-making model fitted in this paper. Functions are shown for different values of parameters *s* and *k* in the model by Arganda et al. (2012). Higher values of *s* form a stronger threshold value, whereas lower values of *s* result in responses similar to linear gradients. Lower values of *k* shift the probability curve left, and create a larger region of indifference between two sites (in this case creating an area with an equal probability of choosing either site at proportions from 0.3 to 0.7). Values of *a* > 1 result in a higher penalty for low-density sites. One important feature of this model is that the probability of picking a site with no individuals is never 0.

In this model, individuals make decisions based on Bayesian estimation, using information generated by others. The derivation of the model introduces a parameter *s* which equates to an individual's judgement that others make a ‘good choice’ *s* times more often than a bad choice. Thus, if *B* is public or social information, then *s* can be considered the rate of social information use. A value of *s* *=* *1* suggests no socially mediated response, or an equal probability of picking any feeder regardless of where individuals are located (*P*(*X*_*i*_) = 0.25 for all four feeders at all times). When *s* *>* *1*, individual decisions are influenced by the distribution of others within the patch (Fig. 2). At small values of *s*, the probability curve is almost linear with only a small increase in the probability of choosing a busy feeder over an empty feeder (see Fig. 2). At larger values of *s*, this curve becomes sigmoidal; therefore the probability of choosing empty feeders approaches 0 and the probability of choosing busy feeders approaches 1.

The second parameter in this model, *k*, estimates the influence of individuals at a feeder on the quality estimate of other feeders. High values of *k* indicate that the relative difference in the proportion of individuals is used (by choosing one option, an individual reduces the estimated quality of all other options for the following individual), whereas low values of *k* suggest that individuals use probability matching based on the absolute number of individuals on each feeder rather than their relative difference. Biologically, values of *k* < 1 suggest a lower threshold of attraction to sites, which may represent birds occasionally choosing slightly less populous sites when the number of individuals in the patch is high.

The third parameter *a* estimates the quality of the nonsocial information available. If patches vary in quality, then *a* can reflect the different baseline probabilities of choosing each patch. Because all four feeders were identical in our study, we did not fit different values of *a* for each feeder. In this case, *a* > 1 results in an increased avoidance of sites with few individuals, and an increased attraction to sites with many individuals (see Fig. 2).

Finally, given that $\sum_{i}P\left(X_{i}|B\right)$ can exceed 1, an additional step of probability matching is used, where each probability is divided by the sum of the probabilities (Arganda et al., 2012). Probability matching may be important in animal decision making, and was shown to be particularly important in the context of animals dynamically switching between locations (Houston & Mcnamara, 1987).

The sigmoidal property of the model has several benefits. First, it provides a flexible response that can be either linear or nonlinear. Second, it makes this model qualitatively compatible with models of predator avoidance in space. For example, in models of the selfish herd, a sigmoidal function best replicated the patterns of groups observed in space (Beecham and Farnsworth, 1999, Viscido et al., 2002). Finally, this model was set within the context of information use, which can refer to food quality or information about predation risk (Arganda et al., 2012, Pérez-Escudero et al., 2013), or be a result of local enhancement and social learning known to occur in this population (Aplin, Sheldon, et al., 2013). However, in our study we quantified model parameters primarily to assess the relative contribution of different components within flocks on individual movements. Thus, although the parameters themselves have biological meaning, we focus here on the similarity or differences of the value of rates of social information use (*s*) for conspecifics and heterospecifics.

To estimate the values of *s*, *k* and *a* in the model, we fitted the model to our data using maximum-likelihood estimation. This was done by calculating the probability of each movement decision (a departure and an arrival) that was observed in the data, based on the values of the parameters. The best-fitting parameters were those with the minimum sum of the log-likelihoods of all decisions combined. We used the mle function to perform this computation in R (R Development Core Team, 2013), and the confint function to estimate the 95% confidence intervals from the log-likelihood profiles.

To calculate the relative weighting applied to the conspecifics and heterospecifics in within-patch foraging decisions, we fitted alternative forms of the decision-making models. These differentiate conspecific versus heterospecific attraction by including independent *s* parameters for each, given by:

(3) $$P\left(X_{i}|B\right)=\frac{1}{1+as_{\text{c}}^{-\left(n_{\text{c}x}-k\sum_{i\neq x}n_{\text{c}i}\right)}s_{\text{h}}^{-\left(n_{\text{h}x}-k\sum_{i\neq x}n_{\text{h}i}\right)}}$$

Here, *s*_c_ and *s*_h_ refer to conspecific and heterospecific attraction respectively, *n*_c*x*_ and *n*_h*x*_ are the number of conspecifics and heterospecifics at site *x*, respectively.

Theoretically, the decision-making model could be fitted for each individual, or even with an *s* parameter for each dyad. However, patches were only deployed for a short period (3 days) and sampling was not repeated within sites in order to provide independent replicates. As a result, we did not have enough repeated movements by individuals to estimate individual-level movement rules. Instead, we tested for variation in the weighting of conspecific versus heterospecific attraction by fitting the *s*_c_ and *s*_h_ parameters from equation 3 to decisions made by blue tits and great tits independently (the two most common species in the study).

(5) To compare observed behaviour with that expected from the model alone, we generated artificial simulated data using the decision probabilities given by this model. At each time step in a simulation run, a random flock of birds was created by randomly drawing an observed flock from our observed data. We then randomly selected a bird that either remained at its current feeder or moved to a new feeder based on the decision probabilities predicted by the best-fitting model (calculated using both *s*_c_ and *s*_h_ parameters). This created 1000 simulated flocks each running for 50 decisions. We extracted between-feeder movements, and these were analysed in the same manner as the experimental data. All analyses, calculations, data handling and simulations were conducted in the software programme R (R Development Core Team, 2013).

### Ethical Note

All work was subject to review by the Department of Zoology (University of Oxford) local ethical review committee and adhered to U.K. standard requirements. Birds were caught, ringed and tagged under BTO licence C5714. PIT tags were fully moulded into an 8 mm plastic ring with no protrusions (see Supplementary movie). This work was conducted as part of a large ongoing research project at Wytham Woods.

## Results

### How Do Birds Distribute Themselves in Foraging Patches?

**Figure A2:**
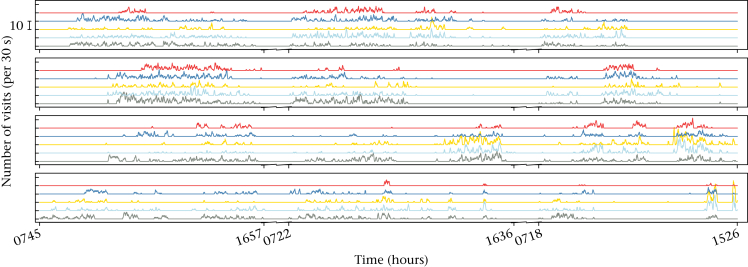
Graphical representation of the data for 3 days (5–7 February 2012) at one replicate of the patch. Each panel is one feeder within a set of four running concurrently. Lines within boxes each represent the feeding activity as the number of visits per 30 s by individuals of each species (top to bottom: marsh tit, nuthatch, great tit, blue tit, coal tit). The lines for each species are displaced vertically in units of 10 for clarity.

In total, we recorded 1904 different tagged individuals (825 blue tits, 813 great tits, 133 marsh tits, 101 coal tits and 32 Eurasian nuthatches). A total of 91 576 feeding visits by these individuals were recorded (34.3% by blue tits, 32.5% by great tits, 16.4% by marsh tits, 11.2% by coal tits and 6.0% by nuthatches). Plots of the raw data within patches showed bursts of synchronized feeding activity within and across species (Appendix Fig. A2). Previous analyses of data collected in this system have shown that these bursts of activity reflect patch visits by flocks of tits (Psorakis, Roberts, Rezek, & Sheldon, 2012).

**Figure 3:**
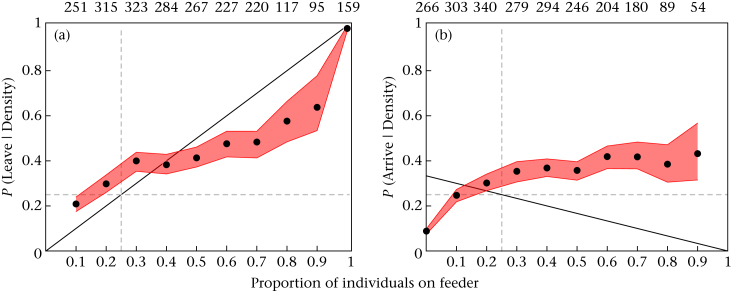
Within-patch movements with respect to distribution of birds across the four feeders. Circles represent the observed probability of a moving bird (a) leaving and (b) arriving given the proportion of individuals present on each feeder. Feeder densities (proportions) were calculated using the number of birds present at each feeder divided by the number present in the whole patch. The shaded envelopes are the maximal variability range from 1000 jackknife estimations with 40% of the original data removed. The vertical dashed lines represent the mean expected density on each feeder (0.25) in the absence of any collective behaviour. The horizontal dashed lines represent a null model based on random selection of leaving and arriving at feeders (selecting a random feeder = 0.25). The solid black lines indicate the expected relationship based on the theoretical asocial prediction (TASP). Values above each plot give the sample size (*n* departures or arrivals) for each data point below it.

**Figure A3:**
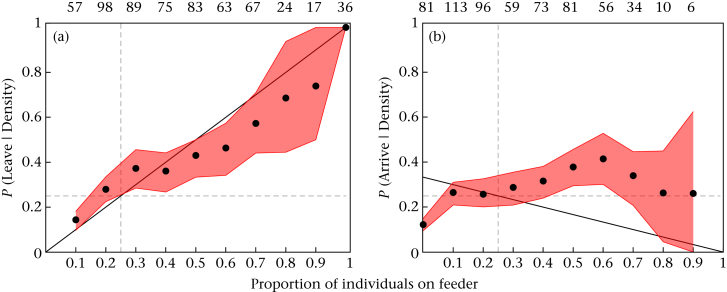
Within-patch movements with respect to distribution of birds across the four feeders for day 1 only. Circles represent observed probability of moving given the proportion of individuals at the feeder when (a) leaving and (b) arriving. Feeder densities (proportions) were calculated using the number of birds present at each feeder divided by the number present in the whole patch. The shaded envelopes are the maximal variability range from 1000 jackknife estimations with 40% of the original data removed. The horizontal and vertical dashed lines represent mean/random expected density on each feeder (0.25) in the absence of any collective behaviour. The solid black lines indicate the density-dependent expectation of the theoretical asocial prediction (TASP). Values above each plot give the sample size (*n* departures or arrivals) for each data point below it.

We detected 2259 within-patch movements by 1138 individuals (21.6% by blue tits, 20.4% by great tits, 17.9% by coal tits, 31.2% by marsh tits, 8.9% by nuthatches). We found that although the probability of leaving sites increased with density, birds were disproportionally more likely to leave low-density sites than high-density sites than if decisions had been made at random (Fig. 3a). Movements were also increasingly likely to be relocations to a feeder with a high density of birds than one with a low density (Fig. 3b). This pattern differed markedly from either a random choice null expectation, or a null model based on avoidance of others (the 95% confidence intervals of our data differ from the theoretical model in Fig. 3). When the data were restricted to the first day of each replicate, a similar pattern was observed (Appendix Fig. A3), confirming that the observed patterns are not the result of changing patch quality over time. Taken together, these results suggest that birds were actively reducing their relative distance to others.

### Do Birds Use a Simple Flocking Rule?

Although we found evidence for universal movement away from low-density parts of the patch, it is possible that attraction to conspecifics and heterospecifics varied by species. Fitting the parameters of the decision-making model (given by equation 3) suggests that great tits relied more heavily on the decisions of conspecifics than heterospecifics (*s*_c_ = 12.64, 95% range 5.05–21.98, *s*_h_ = 2.10, 95% range 2.10–5.17, *a* = 13.48, 95% range 7.38–24.43, *k* = 0.01, 95% range 0–0.02). In contrast, the relative size of the parameter estimates in blue tits were reversed (potentially suggesting greater attraction or information use from heterospecifics), but the substantial overlap in confidence intervals does not support a significant difference in their response to conspecifics and heterospecifics (*s*_c_ = 3.63, 95% range 2.10–9.11, *s*_h_ = 4.05, 95% range 2.69–8.42, *a* = 9.99, 95% range 4.83–21.87, *k* = 0.02, 95% range 0–0.05).

### Can a Simple Rule Replicate Mixed-species Flocking Dynamics?

**Figure 4:**
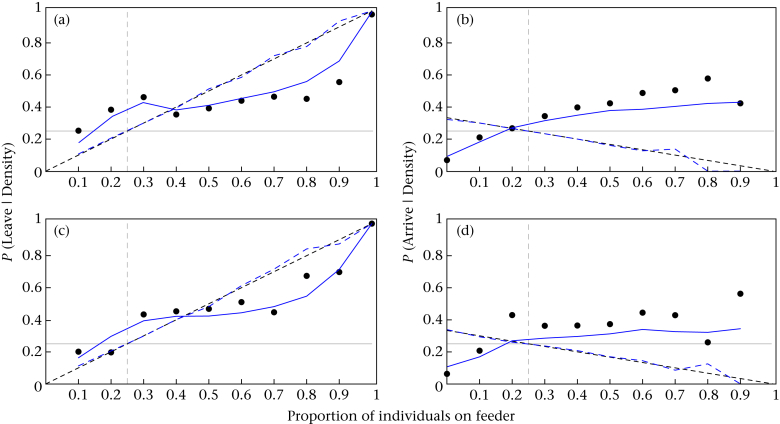
Simulated movements of birds moving between four feeders. Circles represent results from the observed movement of (a, b) blue tits and (c, d) great tits. The solid lines represent the results of simulating birds (a, c) leaving and (b, d) arriving at feeders based on the best-fitting decision rule (see text). The dashed lines represent movements simulated with no decision rules, which exactly replicate the theoretical asocial prediction (TASP, solid black lines). The horizontal and vertical dashed lines are described in Fig. 3.

**Figure 5:**
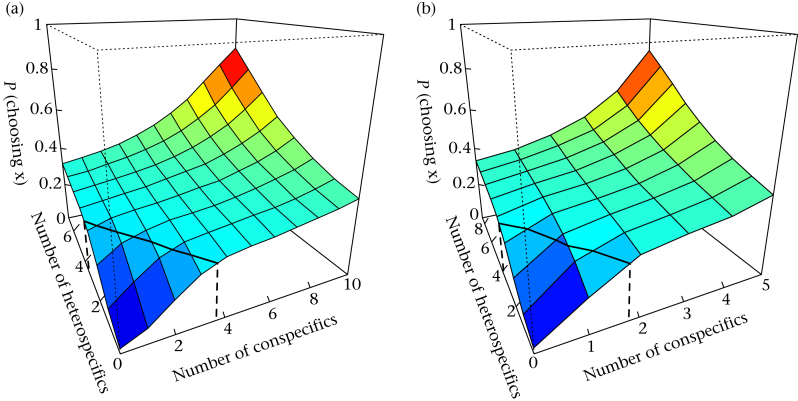
Predicted surface illustrating the dependence of *P* (choosing x) on different combinations of conspecifics and heterospecifics on feeder x for (a) blue tits and (b) great tits. The surface is plotted for the mean group size experienced by individuals of each species, which is shown by the extent of the axes. This remains fixed in order to determine how the probability of choosing x varies according to the distribution of individuals on feeder x as opposed to the rest of the patch. The black line represents the contour of *P* = 0.25, or the threshold above which individuals choose a site more often than at random. The values at which this probability threshold is reached (shown by the dotted black lines) do not stay constant as the flock size changes, but scale proportionately (see Appendix Fig. A4).

Our agent-based simulations of birds making decisions based on the proportion of individuals at each feeder replicated the movement data well (Fig. 4). Movement decisions based on the null value of *s* = 1 also perfectly replicated our theoretical asocial prediction (TASP). Figure 4 also suggests that the resulting responses for great tits and blue tits were very similar. Plotting the probability of choosing a site (based on the model) for different combinations of conspecifics and heterospecifics suggests that despite the differences in parameter estimates, differences in relative abundance led to broadly similar behaviour when birds responded to the flock as a whole (Fig. 5).

**Figure A4:**
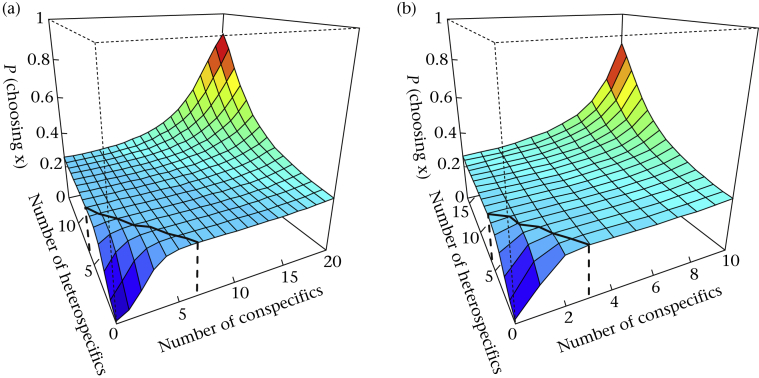
Probability surface of *P* (choosing x) for different combinations of conspecifics and heterospecifics on feeder x for (a) blue tits and (b) great tits. The curve is plotted for twice the mean group size experienced by individuals of each species (see Fig. 4). The black line represents the contour of *P* = 0.25, or the threshold above which individuals choose x more than at random. This shows that the values at which this probability threshold is reached (shown by the dotted black lines) does not stay constant as the flock size changes, but scales proportionately, where birds avoid sites below one-third the number of conspecifics and sites below one-half the number of heterospecifics.

Importantly, the rules we inferred from our data suggest that the probability of an individual choosing a site was less than even (*P*(*X*_*i*_) < 0.25) if fewer than one-third of the conspecifics were present, or sites with fewer than half of the heterospecifics present. For example, blue tits experienced a mean group size of 10 conspecifics, and the decision rule we inferred suggests that they avoided sites with fewer than three or four conspecifics (Fig. 5a). However if group size increased to 20, then the proportion remained the same, rather than the absolute threshold (six or seven individuals, Appendix Fig. A4a). The rule used by great tits showed a similar response, predicting that individuals should choose sites with one or more individuals when there are five conspecifics present (Fig. 5a), and three or more when there are 10 conspecifics (Appendix Fig. A4b). Thus, the proportion, rather than the absolute number of individuals, was consistent for different flock sizes, which clearly reflects the higher-than-expected rate of departure from sites with a density below 0.3 in Fig. 3a.

## Discussion

Our study used automated monitoring of foraging decisions in a wild bird population to quantify aspects of the decision-making processes of wild birds in mixed-species flocks. First, we showed that individual birds foraging within mixed-species flocks actively moved to areas of foraging patches with higher densities of individuals. Our results suggest that coordinated social foraging behaviour in these species was predicted by a rule of attraction towards others. However, we found that this decision-making rule was not applied equally to conspecifics and heterospecifics, nor did individuals of different species have the same weighting for conspecific and heterospecific information use. Despite these differences in the inferred decision rules, we found rather similar behaviour at the flock level for the two numerically dominant species: this similarity seems to result from differences in mean abundance for these two species. Hence, it is possible that different social interaction rules at the species level may arise as an adjustment to species composition, with the result that coherent across-species behaviour is generated. These findings highlight the potential value of applying collective decision-making models to mixed-species groups.

The high values of *s* (term representing social information use) inferred from the data suggest a strong response by individuals to both conspecifics and heterospecifics. This implies that the relative probability of moving towards dense parts of the flock is much higher than the probability of moving towards relative emptiness. This is analogous with a ‘locally crowded horizon’ rule used for modelling selfish herds (Viscido et al., 2002). The *k* parameter, whether individuals use relative or absolute differences, may also be biologically important. Here the value of *k* was relatively low, which suggests individuals were often moving to feeders with a medium number of individuals, as well as feeders containing the most birds. This function allows two sites of medium density to have an equal probability of being chosen even if they differ slightly in the number of individuals present (Fig. 2). This could reflect the variable group sizes we observed and result from effects of competition. When large groups were present, individuals may have favoured movements towards areas of medium density, thereby gaining a balance between antipredation benefits and competition. Alternatively, it may reflect an overall tendency to avoid low-density sites, which is supported by a large value of *a*. Values of *a* > 1 have a large influence on empty sites by reducing their maximum baseline probability (below 1/*N* sites after probability matching is applied). The combination of these forces is considered crucial in the formation of the group size distributions observed in nature (Beecham & Farnsworth, 1999); it may be an important process preventing continuous aggregation of individuals into one increasingly large group.

Although we found similar parameter values to previous studies on fish in captivity (Arganda et al., 2012, Mann et al., 2014, Pérez-Escudero and Polavieja, 2011, Pérez-Escudero et al., 2013), our simulation results suggest that individuals may make decisions based on estimated proportions of individuals across the patch, as opposed to the absolute number. For example, Arganda et al. (2012) found that the rule inferred for a fixed group size in fish was to ‘stop counting above three’ individuals in a patch. However, where the group size varied, our rule seemed to generalize to ‘avoid sites below one-third’ for conspecifics and ‘avoid sites below one-half’ for heterospecifics. This may be an important finding in the context of animal decision making that previous studies were unable to uncover because laboratory experiments are typically performed on fixed group sizes. Further, although we inferred different parameter values for blue tits and great tits, we found that the outcome of their decision-making process was surprisingly similar. This suggests that birds may be adapting their decision-making rule to their local social environment in order to generate a similar response across species.

Our finding that birds used rules that scale proportionally with group size may also reflect a difficulty for animals in estimating how many individuals are performing each behaviour. It is likely that the tits in our study may simply be estimating feeder quality based on the relative rate at which each behaviour is being performed. Numerous studies on patch choice have found that when choosing between different foraging patches, birds typically estimate site quality from the intake rate rather than the absolute number of conspecifics present (reviewed in Stephens et al., 2007). This allows animals to make consistent decisions across different group sizes, and to flexibly adapt to different patch qualities (Stephens, 2008). Our simulations suggest that it is likely that a similar rule applies to birds making movement decisions within flocks as they do between flocks.

The *s* parameter in the decision-making model we used is broadly defined as social information use. This same model was used in an elegant experiment recently performed on fishes by Miller et al. (Miller et al., 2013, Pérez-Escudero et al., 2013) that suggested risk minimization and social information mechanisms are interlinked, and that both contribute to individual decisions. Thus, by exploiting social information in its broadest definition (basing decisions on the behaviour of others or being attracted to popular choices), animals could be using a general rule that satisfies combined needs to reduce risk, such as through dilution, and gain information about the environment, such as finding the best sites in which to forage. Subsequently, social aggregations (sensu Hamilton, 1971) may simply be an emergent social property of an individual-level prioritization of social over personal information (Chamley, 2003). Using this rule, if perceived predation risk goes up, individuals simply increase the attraction parameter of their social rule, driving the group closer together via social reinforcement (Ame et al., 2006). Varying this single parameter in response to ecological conditions may represent a simple mechanism underlying fission–fusion dynamics in the study species (Farine, 2013b).

In summary, our study provides a significant advance in our understanding of social behaviour of mixed-species flocks. Previous studies (for example Jolles et al., 2013) have typically been unable to characterize individual-level decision making, as this requires both (1) individuals to be individually marked and (2) the behaviour of all other members of the flock to be quantified when decisions are made. Further, successfully replicating our data using simulated flocks has enabled us to describe a candidate model for exploring the interaction between forces of selection (such as predation or competition) and behavioural rules. We predict that this result should be generally applicable across a wide range of animals forming mixed-species aggregations. However, wild environments are dynamic and uncertain, with shifting levels of predation and resource availability. Understanding how individuals adjust collective decision-making rules to conspecifics and heterospecifics over changing social and environmental gradients may be a powerful approach for investigating the adaptive value of group living.

## Supplementary Material

Supplementary movie. Video demonstrating access to, and behaviour at, feeders by PIT-tagged birds. Tags fitted to the leg of each bird are read by RFID antennae moulded into the (black) perches. Birds were fed unhusked sunflower seed, and typically flew into a nearby tree to process the food items.
